
JOURNAL INFORMATION
==============================
NLM Title Abbreviation: Gastroenterol Rep (Oxf)
Journal ID: Gastroenterol Rep (Oxf)
NLM Journal ID: 101620508
Journal ID: gastro

Gastroenterology Report

EISSN: 2052-0034
Publisher: Oxford University Press

ARTICLE INFORMATION
==============================
PMCID: PMC5444254
DOI: 10.1093/gastro/gox003
Article ID: gox003
Article version: 1
Subjects: Chinese Abstracts

Chinese Abstracts – Volume 5 Issue 1*

* This article abstract was originally published in English. This translation has not been verified and should not be relied upon—it is provided for reference purposes only. The Publishers have not checked this translation and accept no liability for completeness or accuracy of this translation or the use of this translation for whatever purpose. This translation may be incomplete and inaccurate in whole or in part. If you need to rely upon a translation of this abstract, a professional human translator should be engaged to supply an accurate translation of the original English. When referencing articles from this journal, please always refer to the original English version, rather than a translated equivalent.

Print publication date: 2017 Feb
Electronic publication date: 2017 Feb 7
Volume: 5
Issue: 1
First page: C1
Last page: C3
Copyright: © The Author(s) 2017. Published by Oxford University Press and Sixth Affiliated Hospital of Sun Yat-Sen University
Copyright year: 2017
License: This is an Open Access article distributed under the terms of the Creative Commons Attribution License (http://creativecommons.org/licenses/by/4.0/), which permits unrestricted reuse, distribution, and reproduction in any medium, provided the original work is properly cited.
License URL: https://creativecommons.org/licenses/by/4.0/

Page count: 3

==============================
Gastroenterology Report, 2017, 1–3

doi: 10.1093/gastro/gox003

### Chinese Abstracts – Volume 5 Issue 1*

### Review （综述）

### Re-appraisal and consideration of minimally invasive surgery in colorectal cancer
### 结直肠癌微创手术的重新评价与思考

[摘要] 纵观整个外科史，外科医师一直在努力优化外科术式，减少手术创伤。过去30年间，随着新技术和新术式的开展，结直肠微创手术取得了巨大的进展。腹腔镜手术已显示出等同于甚至优于开放术式的治疗效果。而且，腹腔镜手术已经突破了传统的界限，衍生出单孔腹腔镜、经自然腔道内镜手术及机器人手术等新的微创术式。本文回顾了微创手术的发展历程，并对目前各种结直肠微创术式进行了评价。

关键词：结肠癌；直肠癌；微创手术；腹腔镜；机器人手术；经肛全直肠系膜切除术；经自然腔道标本取出

### Irritable bowel syndrome and diet
### 肠易激综合征与饮食干预

[摘要] 肠易激综合征（IBS）系胃肠道慢性功能性疾病，是最常见的胃肠疾病之一。由于 IBS 患病率高、治疗效果欠佳、经济负担显著，其对一般人群的影响甚大。IBS 的病理生理十分复杂，其治疗主要是对症治疗。对于 IBS 最常采用的治疗方式包括教育和安抚、生活方式的调整（尤其是营养干预）、外周药物（一般是针对肠道动力）、中枢药物（针对内脏高敏性和疼痛）及心理干预（旨在减少应激和症状引起的焦虑）。有效的饮食调节可能包括以下措施：低FODMAPs（发酵的低聚糖、单糖、二糖及多元醇）饮食，限制或禁食产气食物和/或乳糖和麸类食物，补充纤维素。一些新的治疗药物，如某些保健品，也可以作为IBS患者的一项治疗选择。本文重点探讨IBS的饮食干预，对基于营养的饮食治疗的循证医学证据进行了综述。

关键词：肠易激综合征；功能性胃肠疾病；FODMAPs；耐受性；肠道微生态

### Current status of intestinal and multivisceral transplantation
### 肠道移植和多脏器联合移植的现状

[摘要] 临床营养自给是肠衰竭患者的最终目标。一般来说，对于肠衰竭患者，当旨在恢复肠道功能的各种外科和药物治疗努力都失败后，只能接受终身肠外营养。然而在过去20年中，肠道移植方面的研究进展为肠康复治疗体系增加了一个治疗选择。随着在外科技术的标准化、免疫抑制治疗和诱导方案的改进、术后处理措施的改善等方面的不懈努力，肠道移植现已成为可能。肠道移植分为小肠移植、肝-小肠联合移植、多脏器联合移植和改良多脏器联合移植四种方式。目前的指南将肠道移植对象限定于那些具有严重肠外营养并发症（如肝衰竭和反复感染）的患者。随着20世纪90年代他克莫司（一种免抑制药）的应用，无论是在实验研究阶段还是临床实践中，肠衰竭患者肠道移植的疗效都得到了显著改善。研究显示，与肠外营养相比，肠道移植后若能保持13年的肠道功能，其成本效应显著。鉴于肠道移植可延长生存期、改善生活质量、恢复经口进食，肠道移植理应成为肠衰竭患者的一项重要治疗选择。下一步有必要就早期排斥反应标志物的确立、免疫抑制治疗方案的强化、术后康复措施的改善以及是否需早期转移植中心进行治疗等方面展开研究。

关键词：肠道移植；多脏器联合移植；肠外营养；肠衰竭；肠康复

### Original article （论著）

### Postoperative excessive gain in visceral adipose tissue as well as body mass index are associated with adverse outcomes of an ileal pouch
### 术后内脏脂肪组织及体质指数过度增加会引起回肠储袋不良结局

[摘要] 背景：全结直肠切除、回肠储袋肛管吻合（IPAA）术后，内脏脂肪组织（VAT）的变化对于预后的影响，目前尚无相关报道。本项回顾性队列研究旨在评估VAT过度增加对行IPAA术的炎性肠病（IBD）患者临床结局的影响。

方法：收集2002－2014年间我们的前瞻性储袋炎登记病例中，储袋手术后至少接受了2次序贯CT检查的患者。通过CT图像分析计算内脏脂肪面积（VFA）。将VAF增加>15%者归入研究组，VAF增长不足15%者作为对照组。储袋不良结局定义为在初次CT检查后出现储袋慢性炎症进展（慢性储袋炎、慢性封套炎或储袋克罗恩病）、储袋窦道形成或储袋失败，前两项统称为储袋混合事件。

结果：在1564例登记病例中，59例（3.8%）在储袋手术后接受了至少2次CT检查，其中29例（49.2%）归入研究组，30例（50.8%）作为对照组。全组病例初次CT检查至最近一次CT检查的中位间隔时间为552天（312～598天）。两组患者储袋慢性炎症进展发生率分别为13.8% *vs* 3.3%（$p = 0.195$），储袋窦道形成发生率分别为10.3% *vs* 0%（$P =$

*This article abstract was originally published in English. This translation has not been verified and should not be relied upon—it is provided for reference purposes only. The Publishers have not checked this translation and accept no liability for completeness or accuracy of this translation or the use of this translation for whatever purpose. This translation may be incomplete and inaccurate in whole or in part. If you need to rely upon a translation of this abstract, a professional human translator should be engaged to supply an accurate translation of the original English. When referencing articles from this journal, please always refer to the original English version, rather than a translated equivalent.
本文摘要最初是以英文发表。中文摘译未获认证，谨供参考。出版社没有对中文摘译进行核验，不负责译文的完整性和准确性，也不会将其用以其他目的。中文摘译可能会出现部分不完整或不准确的情况。如果你需要准确获悉摘要，最好能请一位专业译者针对英文原文进行翻译。阅读杂志文章，请参考英文原文，不推荐中文翻译。

0.112），储袋混合事件发生率分别为24.1% *vs* 3.3%（$P=0.026$），储袋失败率分别为10.3% *vs* 6.7%（$P=0.671$）。Kaplan-Meier曲线显示，体质指数（BMI）过度增加（>10%）患者无储袋失败生存时间明显缩短（$P=0.011$）。多因素逐步回归分析显示，VAT过度增加是储袋不良结局的独立危险因素（OR＝12.608, 95%CI:1.190～133.538, $P=0.035$）。

结论：在本组回肠储袋患者队列中，储袋手术后VAT或BMI的过度增加长远来看都可能导致储袋不良结局。

关键词：炎性肠病；回肠储袋；储袋失败；内脏脂肪组织；内脏脂肪面积

### Nationwide trends of hospital admissions for acute cholecystitis in the United States
### 美国急性胆囊炎住院情况的趋势分析

[摘要] 背景与目的：急性胆囊炎在胃肠科住院患者中相当常见。本研究的目的是应用美国国家医院数据库来评估急性胆囊炎住院率及住院花费。

方法：我们分析了国家住院病例数据库（NIS），筛选出1997－2012年间将急性胆囊炎（ICD-9代码：574.00、574.01、574.30、574.31、574.60、574.61或575.0）作为第一出院诊断的所有病例。NIS是美国最大的住院数据库，囊括了每年大概8百万患者的住院资料。采用卡方趋势检查分析整个研究期间急性胆囊炎的出院人数、住院时间及住院费用。

结果：1997年有149 661例以急性胆囊炎作为出院第一诊断的住院患者，2012年该人数上升至215 995例（$P<0.001$）。急性胆囊炎的平均住院时间在1997－2012年间减少了17%，由4.7天缩短至3.9天（$P<0.05$）。然而，住院费用却增长了195.4%，由1997年的14 608美元增加至2012年的43 152美元（$P<0.001$）。

结论：在过去16年间，美国急性胆囊炎患者出院人数和住院费用亦显著增加，但平均住院时间却逐渐缩短。急性胆囊炎的住院费用加重了整体医疗支出，有必要进一步评估急性胆囊炎的成本效应和治疗方法。

关键词：住院率；急性胆囊炎；流行病学；趋势

### Esophageal food impaction during cultural holidays and national athletic events
### 文化假日和重大体育赛事期间的食管食物嵌塞

[摘要] 背景：尽管食管食物嵌塞的内在危险因素已经明确，但外在社会行为是否也对会对其产生影响尚未见报道。

方法：我们对2001－2012年间胃肠内镜操作资料软件进行回顾性复习，收集重大体育比赛和法定假日期间在急诊科行食管异物移除的所有病例。

结果：我们发现，在文化假日和重大体育赛事期间行急诊上消化道内镜患者中，食管食物嵌塞者的比例要显著高于非上述特定时期（36.8% *vs* 3.6%, $P<0.001$），增高了10倍。在文化假日/重大体育赛事期间，最常见的嵌塞食物是火鸡（50%），其次是鸡肉（29%）和牛肉（21%）。

结论：食管食物嵌塞常发生于国家假期和重大体育赛事期间，主要嵌塞食物是大块的肉类。建议那些本身就具有食管嵌塞风险的患者，在上述假期或赛事期间，应调整饮食，避免狼吞虎咽和食用大块肉类，以预防食管食物嵌塞。

关键词：食管；异物；食物嵌塞；假期；体育赛事

### Gastric transposition as a valid surgical option for esophageal replacement in pediatric patients: experience from three Italian medical centers
### 胃移位术作为儿童患者食管替代术的有效选择：三家意大利医学中心的经验

[摘要] 背景：食管替代术应用于儿童仅限于很少的情况，包括其他治疗方式无效的长段型食管闭锁和腐蚀性物质摄入导致的食管狭窄。本研究旨在分享三家意大利医学中心胃移位术的应用经验。

方法：这是一项回顾性研究，数据提取自一项前瞻性数据库。我们纳入了该数据库中过去15年所有行胃移位术的儿童患者。

结果：15年期间，8例婴幼儿（男性3例，女性5例）接受了胃移位术。长段型食管闭锁6例，腐蚀性食管狭窄2例。无一例患儿死亡。3例患者出现了早期并发症：2例于术后3周出现自限性涎腺瘘，1例空肠造瘘者出现空肠穿孔，予再次手术治疗。1例患儿出现晚期并发症，即吻合口狭窄，进行了2次内镜扩张。中位随访时间60月（18～144月）。在最后一次临床随访中，6例患儿饮食正常，2例进食困难（原位空肠造瘘）；所有患儿经发音矫正治疗后语言表达良好。所有患儿术后体重和身高均正常增长。

结论：本研究报道了三家意大利中心胃移位术的治疗经验，无论从外科技术（简单、可重复、可接受的并发症）还是从长期随访（良好的经口进食、正常的社会生活、正常的生长曲线）来看，该术式均显示出满意的治疗效果。

关键词：胃移位术；儿童；食管闭锁；腐蚀性物质摄入

### The effect of disc-shaped gastric resection of anastomosis site on reducing postoperative dysphagia and stricture after esophagogastric anastomosis in patients with esophageal cancer
### 盘形胃切除应用于食管胃吻合术改善食管癌患者食术后吞困难和吻合口狭窄

[摘要] 目的：食管切除术仍然是治疗食管癌最可靠的技术，但其吻合口并症包括漏、缺血和狭窄等会影响该术式的治疗效果。本研究旨在评估一种新的食管胃吻合方法能否减少术后吞咽困难和狭窄形成。

方法：80例拟行食管切除术的食管癌患者随机分为研究组与对照组，每组40例。在对照组中，采用线形胃切除进行食管胃吻合；而在研究组中，采用一种新型的盘形胃切除进行吻合。比较两组患者的临床结局。

结果：盘形胃切除组术后吞咽困难和吻合口狭窄的发生率较对照组明显降低（吞咽困难45% *vs* 75%, $P=0.02$；狭窄12.5% *vs* 32.5%, $P=0.03$），而两组患者ICU住院时间、吻合口漏及其他并发症的差异则无统计学意义（均$P>0.05$）

结论：改进外科技术可以减少术后吻合口并发症。本研究中术后吞咽困难和吻合口狭窄的减少可能部分得益于盘形胃切除所提供的大小合适的吻合口。因此，这项新的吻合技术能改善食管切除食管胃吻合患者的临床结局。

关键词：食管癌；食管切除术；食管胃吻合术；术后并发症

### Recurrence and survival rates of inflammatory bowel disease-associated colorectal cancer following postoperative chemotherapy: a comparative study
### 接受术后化疗的炎性肠病相关结直肠癌患者的复发率与生存率：一项比较研究

[摘要]　背景与目的：炎性肠病（IBD）患者具有一定结直肠癌发生风险。研究显示，IBD相关结直肠癌与非IBD相关结直肠癌在肿瘤发生机制和组织形态学上存在差异，提示其肿瘤行为和治疗反应也可能有所不同。我们旨在比较接受术后化疗IBD相关与非IBD相关的结直肠癌患者肿瘤复发及生存率的差异。

方法：检索Cleve?land临床中心结直肠癌数据库，筛选出1994－2010年间的65例IBD相关结直肠癌且接受了术后辅助化疗的患者，排除21例临床资料不完整者。按照年龄、手术目的、肿瘤部位、肿瘤病理分级、AJCC分期和T分期，对IBD相关和非IBD相关的结直肠癌患者进行1:4的病例匹配分组。分别采用竞争风险模型和Cox回归模型来评估IBD对无病生存和总体生存的影响。

结果：44例IBD相关和176例非IBD相关的结直肠癌患者完成匹配。在IBD组患者中，29例（66%）为溃疡性结肠炎，14例（19%）为克罗恩病，1例（2%）诊断为不确定型肠炎。IBD平均诊断年龄为（28.1 ± 14.5）岁，IBD诊断至结直肠癌治疗的平均间期为（21.5 ± 12.6）年。10例（23%）IBD组患者出现了肿瘤复发，而非IBD组则有34例（19%）复发（$P=0.074$）。IBD组与非IBD组患者无瘤生存率（HR＝0.60；95%CI：0.35～1.05；$P=0.074$）和总体生存率（HR＝0.87；95%CI：0.54～1.4；$P=0.58$）的差异均无统计学意义。

结论：接受术后化疗的IBD相关结直肠癌患者，其复发率与生存率与非IBD相关结直肠癌患者相当。有必要行进一步的前瞻性研究来证实这些发现，以指导治疗决策。

关键词：炎性肠病；结直肠癌；辅助化疗；预后

### Serum albumin predicts survival in patients with hilar cholangiocarcinoma

### 血清白蛋白水平预测肝门部胆管癌患者的生存

[摘要] 背景与目的：肝门部胆管癌是一种极具威胁的恶性肿瘤，其发生率随地域及其他危险因素的不同而有所差异，其快速进展的疾病特点及难以早期诊断的现状使相当数量的患者无法得到根治性治疗。本研究的目的是明确影响肝门部胆管癌患者总体生存的预后因素。

方法：收集2003－2013年间经组织学证实的成人肝门部胆管癌患者，评估其预后影响因素，评估变量包括入院时人口学资料、实验室指标、症状及影像学特征。

结果：116例经病理诊断的肝门部胆管癌患者纳入研究。血清白蛋白>3.0 g/dL（$P < 0.01$）、CA19-9 200 U/mL（$P = 0.03$）、癌胚抗原10 μg/L（$P < 0.01$）、有肝硬化病史（$P < 0.01$）或有糖尿病病史（$P = 0.02$）者，其总体生存时间延长。其中血清白蛋白 >3.0 g/dL是一项独立的预后保护因素（HR＝0.31，95%CI：0.14~0.70），可以得到44周的生存获益。

结论：本研究是目前对于肝门部胆管癌预后因素的最大宗病例分析。血清白蛋白 >3.0 g/dL是一项独立的预后指标，可以显著延长生存期。

关键词：肝门部胆管癌；预后因素；生存；白蛋白

### Case Report（病例报告）

### Liver metastasis from hepatoid adenocarcinoma of the esophagus mimicking hepatocellular carcinoma

食管肝样腺癌的肝转移误诊为肝细胞癌

### Diagnosis of primary squamous cell carcinoma of the pancreas using endoscopic ultrasound-guided core needle biopsy

内镜超声引导下针芯活检诊断胰腺原发性鳞状细胞癌

### Ciliated hepatic foregut cyst: report of three cases and review of imaging features

肝脏纤毛前肠囊肿：三例报道和影像学特征复习
